# Association between smoking cessation and post-hospitalization healthcare costs: a matched cohort analysis

**DOI:** 10.1186/s12913-019-4777-7

**Published:** 2019-12-02

**Authors:** Margaret B. Nolan, Bijan J. Borah, James P. Moriarty, David O. Warner

**Affiliations:** 1 Department of Anesthesiology and Perioperative Medicine, Mayo Clinic, 200 First Street SW, Rochester, MN 55905 USA; 2 Mayo Clinic Robert D. and Patricia E. Kern Center for the Science of Health Care Delivery, Mayo Clinic, Rochester, MN USA

**Keywords:** Smoking, Healthcare costs, Smoking cessation, Hospital costs

## Abstract

**Background:**

The potential economic benefit in terms of reduced healthcare costs when patients quit smoking after hospital discharge has not been directly measured. The aim of this study was to compare the costs for hospital admission and six-month follow-up for a cohort of patients who self-reported abstinence from cigarettes at 6 months after hospital discharge and a matched group of patients who reported continued smoking.

**Materials and methods:**

This was a secondary analysis of a recent population-based clinical trial cohort (ClinicalTrials.gov ID: NCT01575145), with cohort membership determined by self-reported 7 day point prevalence abstinence at 6 months after the index hospital discharge. Participants were admitted to Mayo Clinic Hospital, Rochester, MN, between May 5, 2012 and August 10, 2014 for any indication and lived in the areas covered by postal codes included in Olmsted County, MN. Propensity score matching was used to control for differences between groups other than smoking status, and any residual imbalance was adjusted through generalized linear model with gamma distribution for cost and log-link transformation.

**Results:**

Of 600 patients enrolled in the clinical trial, 144 could be contacted and self-reported 7 day point prevalence abstinence at 6 months after hospital discharge. Of these patients, 99 were successfully matched for this analysis. The cost for the index hospitalization was significantly greater in patients who abstained compared to those that did not abstain (mean difference of $3042, higher for abstainers, 95% CI $170 to $5913, *P* = 0.038). However, there was no difference between mean 6-month follow-up costs, number of inpatient hospitalizations, or number of emergency room visits for abstainers versus non-abstainers.

**Conclusion:**

There was no evidence to support the hypothesis that abstinence at 6 months after hospital discharge is associated with a decrease in health care costs or utilization over the first 6 months after hospital discharge.

## Background

An estimated 5 million patients who smoke require admission to US hospitals annually [1]. Hospitalization provides an opportunity to intervene in smoking behavior, and multiple studies have shown that interventions combining counseling with post-discharge follow-up can be efficacious [2], although the dissemination and implementation of such interventions has proved challenging in real-world practice. One of the potential benefits of successful interventions could be a reduction in the costs of medical care after hospital discharge. For example, improvement of smoking-related illness could result in lower readmission rates. Most of the economic analyses regarding the potential impact of smoking cessation on costs rely upon estimates of the costs of smoking-related diseases, and assumptions of how cessation will affect the prevalence of such diseases, rather than direct measurements of costs [3, 4]. In a prior analysis, we demonstrated that smokers undergoing elective surgery incurred the same costs for the index hospitalization compared with never-smokers undergoing the same procedure, but experienced greater costs in the first year after discharge [5]. However, this analysis included only surgical patients and did not include any information regarding smoking status post-discharge, so the potential influence of postoperative abstinence on costs in patients who were smoking at the time of admission could not be measured.

We recently published a controlled trial (ClinicalTrials.gov ID: NCT01575145) in which 600 residents of Olmsted County, MN who were hospitalized at Mayo Clinic Rochester hospitals for any indication were randomized to receive an intervention aimed at facilitating telephone counseling (“quitline”) utilization or a standard brief smoking intervention to explore the efficacy of using telephone quitlines in hospitalized patients to achieve smoking cessation [6]. The quitline facilitation intervention did not change self-reported abstinence rates 6 months after hospital discharge compared with a standard brief intervention (24% in both groups). While there is thus no rationale for analyzing healthcare costs according to treatment assignment, these data do provide the opportunity to explore the association of post-discharge abstinence with healthcare costs in a geographically-defined population across all admission indications.

This secondary analysis of a population-based study compares healthcare costs from hospital admission to 6 months after hospital discharge for those patients in the clinical trial who self-reported abstinence at 6 months and a matched group of trial patients who reported continued smoking. We hypothesized that costs in the 6 month period after discharge would be less in those patients who self-reported abstinence.

## Methods

Both Mayo Clinic and Olmsted Medical Center Institutional Review Boards approved this study.

### Study population

Patients for this analysis were sampled from participants in the randomized clinical trial with whom contact was made at 6 months after hospital discharge to ascertain smoking status. Patients eligible for the trial included those who lived in the areas covered by ZIP codes within Olmsted County, MN (55901–55906, which included all residents of Olmsted County and some who resided just outside of the county), were hospitalized in Mayo Clinic, Rochester between May 5, 2012 and August 10, 2014 for any admission indication, and reported current smoking at the time of admission. All indications were included to examine the association of abstinence with post-hospitalization costs within all members of a geographically-defined population. In conformity with Minnesota statutes, patients that did not authorize the use of their medical record for research were excluded from the analysis.

### Data sources

Data for the study patients including demographic and clinical characteristics were collected from electronic databases. Admission diagnosis was categorized using Clinical Classification Software (CCS) categories based on the primary diagnosis [7]. The Rochester Epidemiology Project Cost Data Warehouse was used to capture standardized cost data for the services that the patients received [8]. This includes costs for services provided by the two healthcare systems serving local residents, the Mayo Clinic and Olmsted Medical Center. In essence, the standardized cost data were estimated by adopting a hybrid approach in which Medicare reimbursement rates were assigned to all professional billed services, while the appropriate cost-to-charge ratios from the Medicare Cost Report were multiplied by the charges for all hospital-billed services [9].

### Statistical analysis

Propensity score matching was conducted to select the non-abstinent cohort based on baseline characteristics including age, gender, and clinical indication based on CCS. The propensity score model used logistic regression with a one-to-one matching approach without replacement. Patients were matched using the nearest neighbor method with a 0.001 caliper. Common support was implemented. Pre- and post-match balance was assessed using the t-test for continuous covariates, and chi-square tests for categorical covariates. Any residual imbalance following propensity score matching was adjusted through generalized linear modeling (GLM). Both index hospitalization and 6-month post-discharge costs were reported for the abstinent and non-abstinent cohorts, along with the mean difference in costs. In an additional analysis, 6-month post-discharge costs were adjusted for index hospitalization costs. All GLM models for cost analyses assumed a gamma distribution for cost and a log-link to account for the skewed nature of the cost costs distribution [10]. Predicted differences in costs between the two groups were estimated. Statistical difference was determined using the 95% confidence intervals (CI) of the difference of the mean predicted costs. Assessment was also performed regarding whether the abstinent and non-abstinent patients differed in terms of their rate of readmission or emergency room (ER) admission during the 6-month follow-up following discharge from the index hospitalization. Post-discharge healthcare utilization in the 6-month follow-up was also characterized in the abstinent and non-abstinent cohorts using Berenson-Eggers Types of Service (BETOS) categories [11], which includes evaluation and management, procedures, imaging, tests, durable medical equipment, others and unclassified. Propensity score matching and other analyses were performed on Stata Software (version 15.1, Statacorp).

## Results

Of the 600 patients enrolled in the clinical trial, 144 could be contacted and self-reported 7 day point prevalence abstinence at 6 months after hospital discharge. For 99 of these patients, it was possible to find an acceptable match among trial patients who could be contacted and reported current smoking. Even with this selection, propensity score matching could not completely balance the two cohorts in terms of their clinical indication. Therefore, this was included as a simple covariate in the cost regressions. Pre- and post- matching balance is given in Additional file 1. The mean age of those included in this analysis was 47.5 years. Post-matching *p*-values showed no statistical difference in covariates and thus good balance. Other variables not included in the propensity matching, including marital status, insurance status, receipt of surgery during admission, and comorbidity counts were compared between abstainers and non-abstainers and no significant differences were found (Table 1). Therefore, 6 month follow-up cost comparisons were performed on unadjusted costs.

**Table 1:** Subject demographics

	Abstainers^a^ (*N* = 99)	Non-abstainers (N = 99)	*p* value
Marital status			0.23
Divorces	25 (25.3%)	25 (25.3%)	
Married	30 (30.3%)	29 (29.3%)	
Separated	4 (4.0%)	0 (0.0%)	
Single	37 (37.4%)	38 (38.4%)	
Widowed	3 (3.0%)	7 (7.1%)	
Insurance status			0.13
Government	52 (52.5%)	63 (63.6%)	
No insurance	5 (5.1%)	4 (4.0%)	
Private	38 (38.4%)	32 (32.3%)	
Self pay	4 (4.0%)	0 (0.0%)	
Surgery during hospitalization			0.65
No	63 (63.6%)	66 (66.7%)	
Yes	36 (36.4%)	33 (33.3%)	
Count of Elixhauser comorbidities			0.90
Mean (SD)	2.7 (1.9)	2.8 (2.0)	
Median	2.0	3.0	
Q1, Q3	1.0, 4.0	1.0, 4.0	
Range	(0.0–9.0)	(0.0–11.0)	

^a^Self-reported abstinence at 6 months after hospital discharge

The cost of index hospitalization were significantly greater in patients who abstained compared to those that did not abstain (mean difference in costs of $3042, higher for abstainers, 95% CI $170 to $5913, *P* = 0.038) (Table 2). However, the 6-month follow-up costs of abstainers and non-abstainers were not different, with large confidence intervals for the estimated differences ($4196 higher in abstainers, 95% CI -$6301 to $14,693, *p* = 0.433) (Table 2). Adjusting the 6-month costs for the cost of the index hospitalization had little effect on this finding. There was no significant difference in the number of inpatient hospitalizations or emergency room visits between abstainers and non-abstainers in the 6 months after hospital discharge (Table 3)**.** When utilization categories of follow-up were broken down further into categories such as imaging, evaluation and management, and procedures, there were also no differences in any category between abstainers and non-abstainers (Table 4). A full listing of diagnostic categories for the index hospitalization of abstainers vs. non-abstainers is presented in Additional file 2. When sorted by type of diagnostic category, the mean cost of initial hospitalization for abstainers was higher in 9 of 14 categories and median cost for abstainers was higher in 7 of 14 categories compared to non-abstainers (Additional file 3). A similar pattern of results was observed for the 6-month follow-up costs.

**Table 2:** Index hospitalization and 6-month follow-up costs of propensity -matched abstainers and non-abstainers

	Mean	95% CI	*p*-value
Index hospitalization costs			
Non-abstainers	$12,471.74	$10,818.27 to $14,125.22	
Abstainers^a^	$15,513.28	$13,091.74 to $17,934.83	
Difference	$3041.54	$170.13 to $5912.95	0.038
6-month follow-up costs			
Non-abstainers	$18,789.63	$13,185.05 to $24,394.21	
Abstainers^a^	$22,985.64	$11,004.71 to $34,966.57	
Difference	$4196.01	-$6300.65 to $14,692.68	0.433
6-month follow-up costs, adjusted for index hospitalization costs			
Non-abstainers	$18,759.77	$13,187.24 to $24,332.29	
Abstainers^a^	$23,007.87	$10,943.85 to $35,071.89	
Difference	$4248.10	-$6332.37 to $14,828.58	0.431

Follow-up costs are cumulative since the date of hospital discharge

^a^Self-reported abstinence at 6 months after hospital discharge

**Table 3:** Inpatient readmission and Emergency Room (ER) utilization by abstainers and non-abstainers

	Non-abstainers	Abstainers^a^	*p*-value
No admission	67 (67.7%)	61 (62.9%)	0.48
Admission	32 (32.3%)	36 (37.1%)	
No ER Visit	42 (42.4%)	35 (36.1%)	0.36
ER Visit	57 (57.6%)	62 (63.9%)	

Values are n (%)

^a^Self-reported abstinence at 6 months after hospital discharge

**Table 4:** Utilization in the Berenson-Eggers Types of Service (BETOS) categories

	n	Mean	SD	Median	IQR	Min/Max
Evaluation & management						
Non-abstainers	99	24.0	31.9	13	6, 27	1 / 203
Abstainers^a^	94	20.2	21.9	12.5	4, 29	1 / 94
Durable medical equipment						
Non-abstainers	35	4.5	6.2	2	1, 6	1 / 29
Abstainers^a^	26	2.8	2.9	1	1, 4	1 / 12
Imaging						
Non-abstainers^a^	82	7.9	10.3	5	3, 8	1 / 67
Abstainers^a^	82	7.0	10.2	4	2 / 8	1 / 77
Procedures						
Non-abstainers^a^	75	14.0	23.0	4	3, 13	1 / 109
Abstainers^a^	72	9.6	13.6	5	2, 10.5	1 / 79
Tests						
Non-abstainers	93	15.8	20.2	8	4, 20	1 / 111
Abstainers^a^	91	13.3	17.4	7	3, 18	1 / 93
Unclassified						
Non-abstainers	62	5.2	6.8	3	1, 6	1 / 39
Abstainers^a^	56	6.6	10.3	3	2, 7	1 / 54
Other						
Non-abstainers	83	11.6	17.7	6	2, 11	1 / 107
Abstainers^a^	80	9.3	12.6	5	2, 12	1 / 81

^a^Self-reported abstinence at 6 months after hospital discharge

*SD* Standard deviation, *IQR* Interquartile range, *Min* minimum value, *Max* Maximum value

## Discussion

The major finding of this study is that in a geographically-based cohort of cigarette smokers admitted to a single institution, there was no evidence of an association between self-reported abstinence and health care costs or utilization over the first 6 months after hospital discharge.

Most studies of the incremental healthcare costs associated with smoking utilize models that include the estimated costs of smoking-related diseases and how smoking affects their prevalence. Several studies have used this method to estimate the economic benefits of quitting smoking in patients with medical conditions. Studies of inpatient smoking cessation interventions using this approach suggest significant cost benefits of interventions starting as early as 3 months post-intervention, and virtually all show cost effectiveness by 1 year [12–14]. Similar studies that use this method to estimate the economic benefits of quitting for specific smoking-related diseases, such as myocardial infarction or stroke, also show significant medical cost benefits to quitting smoking starting about 1 year after cessation, which are sustained throughout the study period (several years, in some cases) [12, 13, 15]. However, few studies have attempted to actually measure changes healthcare costs associated with quitting in individual patients. Studies examining the economic impact of quitting ideally would randomize patients to absolutely quit smoking or continue smoking, which would be neither feasible nor ethical. Thus, such studies typically utilize observational designs, which have the potential for confounding. For example, some studies find that quitting in ambulatory populations is actually associated with an *increase* in annual health care costs, which may last for several years before declining [16–19]. The most likely explanation for this seeming paradox is the “teachable moments” effect of illness and hospitalization; those patients who are more ill are more likely to quit. Thus, such increases in healthcare costs may reflect the severity of the underlying illness that prompted quitting, rather than an effect of abstinence itself. Also, although abstinence from cigarettes has immediate physiologic benefits, it may take some time before there are significant improvements (or arrest in the rate of decline) in smoking-related diseases such as chronic obstructive pulmonary disease that could translate to a reduction in costs.

When considering the potential impact of quitting on actual costs, it is also important to distinguish between study designs that compare costs according to smoking status (typically as current, former, and never smokers) and those that assess costs in smokers who do and do not quit. Regarding the former design as applied to hospitalized smokers, a prior analysis that also included smokers who were enrolled in the present clinical trial found no association between smoking status and hospital costs for either medical or surgical patients [20]. In another analysis restricted to surgical patients, costs in the first year after hospitalization were higher in current smokers compared with never smokers; medical patients were not included in this analysis [5]. Another study found a small but significant increase in the cost of hospitalization in surgical patients who were current smokers compared to those who were never or former smokers; post hospitalization costs were not studied [21]. These are interesting analyses, but may not be useful to infer the potential effect of quitting on costs and utilization; costs may well differ in recent quitters compared with never smokers.

To our knowledge, no prior study has compared post hospitalization costs and utilization in patients who did and did not quit smoking after hospitalization, so there are no data directly comparable to our results. The original intent at the time that the randomized trial was planned was to analyze post hospitalization costs according to treatment assignment, hypothesizing that postoperative abstinence rates would differ between groups, and that the economic impact of these differential rates could be analyzed. However, there was no difference in quit rates according to treatment assignment, thus no rationale to perform this originally-planned economic analysis. Nonetheless, these data provided the opportunity to examine the association between quitting and post hospitalization costs, albeit with the same potential limitations inherent in other observational studies. In particular, it is possible that differences in patient characteristics not accounted for in the analysis, such as severity of illness, contribute to the lack of difference in costs between those who did and did not quit. More severe illness may contribute to both the likelihood of quitting and increased costs, obscuring any potential benefit of abstinence on healthcare utilization and costs. This possibility is supported by the finding that the costs of the index hospitalization of the patients that abstained after discharge were higher, as both groups of patients were abstinent while in hospital. It is also possible that differences might have become apparent if subjects were followed for a longer period of time. Thus, these results cannot be used to evaluate the potential economic benefit of a smoking cessation intervention. Nonetheless, we find no evidence that quitting is associated with a decrease in initial post-hospitalization costs. Thus, although there are certainly many good reasons to treat hospitalized patients for tobacco dependence, there is not yet evidence that such treatment is associated with immediate economic benefit. Rather, based on the results of long-term longitudinal studies of costs showing that economic benefits take some time to accrue, it may also take a considerable period of time before similar benefits would be observed in hospitalized patients.

Although there are several strengths of our study design, including the fact that participants were drawn from an area of geographic residence (reducing the potential for referral bias) and a billing resource that allowed for complete ascertainment of costs using a validated methodology, this study has some significant limitations. First, the number of patients available for analysis was relatively small, and, as typical of cost studies, there was large variation in costs among cases. Some of those who abstained were not included in the analysis because a suitable matched patient could not be identified. This limits the ability to detect differences in costs. Second, as with any observational study, there is the potential for confounding factors not captured in our analytic techniques employed to adjust between groups (such as severity of illness, as discussed). The analysis relied on self-reported smoking status at 6 months, as only about half of the patients in the clinical trial provided urine samples for biochemical confirmation [6]. Although the results among those who provided samples was reassuring regarding the general accuracy of self-report, it is possible that not all self-reported abstainers actually were abstaining, which would bias against finding differences in costs if they existed. We ascertained 7-day point prevalence abstinence rather than continuous abstinence, and it is possible that some patients who were abstinent at 6 months had smoked at some time between hospital discharge and this time. However, there is a good correlation between point prevalence and continuous abstinence, and both methods produce similar results when applied to clinical trials [22]. Finally, this analysis includes a single hospital; a multi-center design would be desirable for future studies.

In conclusion, this study found no evidence to support the hypothesis that maintaining abstinence after hospital discharge is associated with a decrease in health care costs or utilization over the first 6 months after hospital discharge. These results may suggest caution in using the incremental costs of smoking-related illnesses to estimate the immediate potential economic impact of smoking cessation in medical populations. However, because this study had several limitations, including relatively small size and relatively large inter-individual differences in in health care costs, larger studies will be needed to more adequately address this important question.

## Supplementary information

**Additional file 1.** Pre- and post-matching balance of age, sex and clinical indication propensity score matching of abstainers and non-abstainers.

**Additional file 2.** Frequency of diagnoses by 6-month abstinence.

**Additional file 3.** Mean and median costs of index hospitalization and 6-month follow-up costs by indication for subjects who continued to smoke versus those who were abstinent at 6-month follow-up, according to diagnostic category.

## Data Availability

This dataset regarding patient costs is proprietary information of the Mayo Clinic and Olmsted Medical Center, and is not publically available.

## Abbreviations

BETOS: Berenson-Eggers Types of Service
CCS: Clinical Classification Software
CI: Confidence intervals
ER: Emergency room
GLM: Generalized linear modeling
MN: Minnesota
