# Supplementary material for: Association between smoking cessation and post-hospitalization healthcare costs: a matched cohort analysis
Source: BMC Health Serv Res. 2019 Dec 2;19:924. doi: 10.1186/s12913-019-4777-7 (PMC6889662; doi:10.1186/s12913-019-4777-7)
Supplement: Supplementary file 1 — Additional file 1. Pre- and post-matching balance of age, sex and clinical indication propensity score matching of abstainers and non-abstainers. [file 12913_2019_4777_MOESM1_ESM.docx]

**Additional File 1.** Pre- and post-matching balance of age, sex and clinical indication propensity score matching of abstainers and non-abstainers

| **Variable** | **Analysis** | **Mean** | | **p-value** |
| --- | --- | --- | --- | --- |
|  |  | **Abstainer*** | **Non-abstainer** |  |
| Age (years) | Pre-matching  Post-matching | 49.3  47.1 | 47.1  47.8 | 0.186  0.711 |
| Female (%) | Pre-matching  Post-matching | 45.1%  45.5% | 52.9%  50.5% | 0.157  0.479 |
| Neoplasm (%) | Pre-matching  Post-matching | 4.1%  3.0% | 3.0%  3.0% | 0.594  1.000 |
| Endocrine, nutritional, and metabolic (%) | Pre-matching  Post-matching | 2.5%  3.0% | 5.7%  3.0% | 0.161  1.000 |
| Disease of the blood (%) | Pre-matching  Post-matching | 0.8%  0.0% | 1.5%  0.0% | 0.573  - |
| Mental illness (%) | Pre-matching  Post-matching | 7.4%  6.1% | 9.9%  11.1% | 0.427  0.207 |
| Diseases of the nervous system (%) | Pre-matching  Post-matching | 2.5%  3.0% | 4.9%  3.0% | 0.257  1.000 |
| Diseases of the circulatory system (%) | Pre-matching  Post-matching | 30.3%  27.3% | 16.7%  26.3% | 0.002  0.873 |
| Diseases of the respiratory system (%) | Pre-matching  Post-matching | 10.7%  11.1% | 7.2%  10.1% | 0.258  0.819 |
| Diseases of the digestive system (%) | Pre-matching  Post-matching | 10.7%  13.1% | 15.6%  9.1% | 0.196  0.368 |
| Diseases of the genitourinary system (%) | Pre-matching  Post-matching | 0.8%  1.0% | 3.0%  1.0% | 0.180  1.000 |
| Complications of pregnancy (%) | Pre-matching  Post-matching | 4.1%  5.1% | 8.4%  5.1% | 0.128  1.000 |
| Diseases of the skin (%) | Pre-matching  Post-matching | 3.3%  4.0% | 4.9%  3.0% | 0.461  0.702 |
| Diseases of the musculoskeletal system (%) | Pre-matching  Post-matching | 4.9%  4.0% | 4.2%  4.0% | 0.745  1.000 |
| Injury and poisoning (%) | Pre-matching  Post-matching | 12.3%  14.1% | 8.4%  14.1% | 0.225  1.000 |
| Symptoms, signs, and ill-defined codes (%) | Pre-matching  Post-matching | 2.5%  1.0% | 1.5%  3.0% | 0.523  0.315 |

*Self-reported abstinence at 6 months after discharge

*Reference category of clinical classification for propensity score model: Infections and parasitic diseases
