# Supplementary material for: Association between smoking cessation and post-hospitalization healthcare costs: a matched cohort analysis
Source: BMC Health Serv Res. 2019 Dec 2;19:924. doi: 10.1186/s12913-019-4777-7 (PMC6889662; doi:10.1186/s12913-019-4777-7)
Supplement: Supplementary file 2 — Additional file 2. Frequency of diagnoses by 6-month abstinence. [file 12913_2019_4777_MOESM2_ESM.docx]

| **Diagnosis**  **Additional File 2**: Frequency of diagnoses by 6-month abstinence | **Non-Abstainer** | **Abstainer** |
| --- | --- | --- |
| **009.0 INFECTIOUS ENTERITIS NOS** | 1 | 0 |
| **009.1 ENTERITIS OF INFECT ORIG** | 0 | 1 |
| **038.42 E. COLI SEPTICEMIA** | 1 | 1 |
| **038.9 SEPTICEMIA NOS** | 3 | 3 |
| **047.9 VIRAL MENINGITIS NOS** | 0 | 1 |
| **141.0 MAL NEO TONGUE BASE** | 0 | 1 |
| **150.5 MAL NEO LOWER 3RD ESOPH** | 1 | 0 |
| **153.4 MALIGNANT NEOPLASM CECUM** | 0 | 1 |
| **162.5 MAL NEO LOWER LOBE LUNG** | 1 | 0 |
| **183.0 MALIGN NEOPL OVARY** | 0 | 1 |
| **250.82 DM MANIF NEC TYP II UNCN** | 0 | 1 |
| **251.2 HYPOGLYCEMIA NOS** | 1 | 0 |
| **276.1 HYPONATREMIA** | 0 | 1 |
| **276.4 MIXED ACID-BASE BAL DIS** | 1 | 0 |
| **276.51 DEHYDRATION** | 0 | 1 |
| **277.31 FAM MEDITERRANEAN FEVER** | 1 | 0 |
| **291.3 ALC-IND PSYCH DIS/HALLUC** | 0 | 1 |
| **291.81 ALCOHOL WITHDRAWAL** | 0 | 2 |
| **292.0 DRUG WITHDRAWAL** | 1 | 0 |
| **295.30 PARANOID SCHIZO-UNSPEC** | 1 | 1 |
| **295.75 SCHIZOAFFECTIVE-REMISS** | 1 | 0 |
| **296.33 RECUR MJR DEPRESS-SEVERE** | 2 | 0 |
| **296.40 BIPOLAR I-REC MANIC NOS** | 1 | 0 |
| **296.89 OTHER BIPOLAR DIS NEC** | 1 | 0 |
| **296.90 EPISODIC MOOD DISORD NOS** | 1 | 0 |
| **303.00 AC ALCOHOL INTOX-UNSPEC** | 0 | 1 |
| **304.40 AMPHETAMIN DEPEND-UNSPEC** | 0 | 1 |
| **305.00 ALCOHOL ABUSE-UNSPEC** | 1 | 0 |
| **338.11 ACUTE PAIN D/T TRAUMA** | 1 | 0 |
| **338.19 OTHER ACUTE PAIN** | 0 | 1 |
| **345.51 FOC EPILEP SIMPL INTRACT** | 1 | 0 |
| **401.9 HYPERTENSION NOS** | 1 | 0 |
| **402.91 HYPERTEN HEART DIS-FAIL** | 1 | 0 |
| **410.11 ANTER AMI NEC-INIT EPISD** | 0 | 1 |
| **410.31 INFEROPOS AMI-INIT EPISD** | 1 | 1 |
| **410.41 INFER AMI NEC-INIT EPISD** | 1 | 2 |
| **410.71 SUBENDO INFRC-INIT EPISD** | 3 | 5 |
| **413.9 ANGINA PECTORIS NEC/NOS** | 1 | 0 |
| **414.00 CORNARY ATHERO-VESL NOS** | 1 | 0 |
| **414.01 CORNARY ATHERO-NATV VESL** | 3 | 5 |
| **414.02 CORNRY ATHER-AUT BYP GFT** | 1 | 0 |
| **424.1 AORTIC VALVE DISORDER** | 1 | 2 |
| **427.31 ATRIAL FIBRILLATION** | 1 | 2 |
| **427.32 ATRIAL FLUTTER** | 1 | 0 |
| **428.31 AC DIASTOL HEART FAIL** | 1 | 0 |
| **428.33 AC-CHR DIASTOL HRT FAIL** | 1 | 0 |
| **430 SUBARACHNOID HEMORRHAGE** | 0 | 1 |
| **432.1 SUBDURAL HEMORRHAGE** | 1 | 0 |
| **433.11 CAROTID ART OCC W INFARC** | 1 | 1 |
| **434.11 CEREBRAL EMBOL W INFARCT** | 0 | 2 |
| **434.91 CEREBR ART OCC W INFARCT** | 0 | 3 |
| **440.21 ATHEROSCLER-LIMB&CLAUDIC** | 0 | 1 |
| **440.22 ATHEROSCL-LIMB&REST PAIN** | 2 | 1 |
| **453.86 AC EMBL INTERNL JUG VEIN** | 1 | 0 |
| **475 PERITONSILLAR ABSCESS** | 1 | 0 |
| **478.19 OTH DIS NASAL CAV& SINUS** | 0 | 1 |
| **482.1 PSEUDOMONAL PNEUMONIA** | 0 | 1 |
| **482.30 STREPTOCOC PNEUMONIA NOS** | 1 | 0 |
| **482.83 GRAM NEG PNEUMONIA NEC** | 0 | 1 |
| **486 PNEUMONIA, ORGANISM NOS** | 1 | 1 |
| **488.02 FLU DT AVIAN W OTH RESP** | 0 | 1 |
| **491.21 OBST CH BRONCH W/ EXAC** | 1 | 1 |
| **492.8 EMPHYSEMA NEC** | 0 | 1 |
| **493.22 CHR OBS ASTHMA W/EXAC** | 2 | 0 |
| **493.92 ASTHMA NOS W/ EXACER** | 0 | 1 |
| **512.1 IATROGENIC PNEUMOTHORAX** | 1 | 1 |
| **512.81 PRIM SPONT PNEUMOTHORAX** | 2 | 0 |
| **512.89 OTHER PNEUMOTHORAX** | 0 | 2 |
| **518.81 ACUTE RESP FAILURE** | 0 | 1 |
| **518.84 ACUTE & CHR RESP FAILURE** | 1 | 0 |
| **530.20 ULCER OF ESOPH W/O BLEED** | 1 | 0 |
| **531.40 CHR STOMACH ULC W HEM** | 1 | 0 |
| **531.90 STOMACH ULCER NOS** | 0 | 1 |
| **536.2 PERSISTENT VOMITING** | 0 | 1 |
| **540.0 AC APPEND W PERITONITIS** | 1 | 0 |
| **552.21 OBSTR INCISIONAL HERNIA** | 0 | 1 |
| **553.1 UMBILICAL HERNIA** | 1 | 0 |
| **555.2 REG ENTERIT SM/LG INTEST** | 0 | 1 |
| **557.9 VASC INSUFF INTEST NOS** | 1 | 0 |
| **558.9 NONINF GASTROENTERIT NEC** | 0 | 1 |
| **560.81 INTESTINAL ADHES W OBSTR** | 0 | 1 |
| **560.89 INTESTINAL OBSTRUCT NEC** | 0 | 1 |
| **562.11 DIVERTICULITIS OF COLON** | 1 | 0 |
| **564.00 CONSTIPATION, UNSPEC** | 1 | 0 |
| **571.2 ALCOHOL CIRRHOSIS LIVER** | 1 | 0 |
| **572.2 HEPATIC ENCEPHALOPATHY** | 0 | 1 |
| **577.0 ACUTE PANCREATITIS** | 1 | 2 |
| **578.0 HEMATEMESIS** | 1 | 0 |
| **578.1 BLOOD IN STOOL** | 0 | 2 |
| **584.9 ACUTE KIDNEY FAILURE NOS** | 0 | 1 |
| **599.0 URIN TRACT INFECTION NOS** | 1 | 0 |
| **644.03 THRT PREM LABOR-ANTEPART** | 1 | 0 |
| **644.21 EARLY ONSET DELIVERY-DEL** | 0 | 1 |
| **648.21 ANEMIA-DELIVERED** | 1 | 0 |
| **648.91 OTH CURR COND-DELIVERED** | 1 | 1 |
| **654.21 PREV C-SECT NOS-DELIVER** | 1 | 1 |
| **659.71 ABN FETAL HRT RATE, DEL** | 1 | 1 |
| **674.54 CARDIOMYOPATH POSTPARTUM** | 0 | 1 |
| **682.2 CELLULITIS OF TRUNK** | 1 | 0 |
| **682.3 CELLULITIS OF ARM** | 0 | 1 |
| **682.4 CELLULITIS OF HAND** | 1 | 0 |
| **682.5 CELLULITIS OF BUTTOCK** | 1 | 0 |
| **682.6 CELLULITIS OF LEG** | 0 | 2 |
| **686.9 LOCAL SKIN INFECTION NOS** | 0 | 1 |
| **715.35 LOC OSTEOARTH NOS-PELVIS** | 1 | 0 |
| **715.36 LOC OSTEOARTH NOS-L/LEG** | 0 | 1 |
| **719.45 JOINT PAIN-PELVIS** | 0 | 1 |
| **721.3 LUMBOSACRAL SPONDYLOSIS** | 1 | 0 |
| **722.10 LUMBAR DISC DISPLACEMENT** | 1 | 1 |
| **724.2 LUMBAGO** | 0 | 1 |
| **727.05 TENOSYNOV HAND/WRIST NEC** | 1 | 0 |
| **780.2 SYNCOPE AND COLLAPSE** | 2 | 0 |
| **780.39 OTHER CONVULSIONS** | 0 | 1 |
| **780.4 DIZZINESS AND GIDDINESS** | 1 | 0 |
| **786.09 RESPIRATORY ABNORM NEC** | 1 | 0 |
| **786.59 CHEST PAIN NEC** | 3 | 0 |
| **789.03 RLQ ABDOMINAL PAIN** | 0 | 1 |
| **789.09 ABDOMINAL PAIN-SITE NEC** | 1 | 0 |
| **805.01 FX C1 VERTEBRA-CLOSED** | 0 | 1 |
| **805.07 FX C7 VERTEBRA-CLOSED** | 1 | 0 |
| **805.4 FX LUMBAR VERTEBRA-CLOSE** | 1 | 0 |
| **810.03 FX CLAVICL, ACROM END-CL** | 0 | 1 |
| **812.00 FX UP END HUMERUS NOS-CL** | 0 | 1 |
| **812.31 FX HUMERUS SHAFT-OPEN** | 1 | 0 |
| **820.8 FX NECK OF FEMUR NOS-CL** | 1 | 0 |
| **823.02 FX UP TIBIA W FIBULA-CL** | 0 | 1 |
| **823.22 FX SHAFT FIB W TIB-CLOS** | 0 | 1 |
| **824.8 FX ANKLE NOS-CLOSED** | 1 | 0 |
| **850.0 CONCUSSION W/O LOC** | 0 | 1 |
| **854.02 BRAIN INJ NEC-BRIEF LOC** | 0 | 1 |
| **854.06 BRAIN INJ NEC-LOC NOS** | 0 | 1 |
| **860.0 TRAUM PNEUMOTHORAX-CLOSE** | 1 | 0 |
| **873.49 OPEN WOUND OF FACE NEC** | 1 | 0 |
| **879.2 OPN WND ANTERIOR ABDOMEN** | 1 | 0 |
| **881.00 OPEN WOUND OF FOREARM** | 0 | 1 |
| **882.1 OPN WOUND HAND-COMPLICAT** | 1 | 0 |
| **890.1 OPEN WND HIP/THIGH-COMPL** | 0 | 1 |
| **975.4 POISONING-ANTITUSSIVES** | 1 | 0 |
| **980.0 TOXIC EFF ETHYL ALCOHOL** | 1 | 0 |
| **996.69 INFECT DUE TO DEVICE NEC** | 1 | 0 |
| **996.72 COMP NEC D/T HRT DEV NEC** | 1 | 1 |
| **996.74 COMP NEC D/T VAS DEV NEC** | 0 | 1 |
| **998.59 OTHER POSTOP INFECTION** | 0 | 1 |
| **V58.11 ENC ANTINEOPLSTC CHEMO** | 1 | 0 |
| **Total** | 99 | 99 |
