# Supplementary material for: Association between smoking cessation and post-hospitalization healthcare costs: a matched cohort analysis
Source: BMC Health Serv Res. 2019 Dec 2;19:924. doi: 10.1186/s12913-019-4777-7 (PMC6889662; doi:10.1186/s12913-019-4777-7)
Supplement: Supplementary file 3 — Additional file 3. Mean and median costs of index hospitalization and 6-month follow-up costs by indication for subjects who continued to smoke versus those who were abstinent at 6-month follow-up, according to diagnostic category. [file 12913_2019_4777_MOESM3_ESM.docx]

| Indication | Abstainer*  N (%) | Non-Abstainer  N (%) | Mean | | | Median | | | |
| --- | --- | --- | --- | --- | --- | --- | --- | --- | --- |
|  |  |  | **All** | **Abstainer*** | **Non-Abstainer** | | **All** | **Abstainer*** | **Non-Abstainer** |
| Other | 1 (25) | 3 (75) | $5,855 | $6,167 | $5,751 | $6,119 | | $6,167 | $6,070 |
| Genitourinary | 1 (50) | 1 (50) | $7,461 | $9,621 | $5,302 | $7,461 | | $9,621 | $5,302 |
| Skin | 4 (57) | 3 (43) | $7,918 | $5,771 | $10,780 | $6,218 | | $5,135 | $8,453 |
| Respiratory | 11 (52) | 10 (48) | $8,251 | $10,892 | $5,346 | $5,244 | | $5,282 | $4,829 |
| Endocrine | 3 (50) | 3 (50) | $9,154 | $9,988 | $8,319 | $8,699 | | $9,171 | $8,228 |
| Mental Illness | 6 (35) | 11 (65) | $9,749 | $7,438 | $11,010 | $8,705 | | $7,121 | $11,156 |
| Digestive | 13 (59) | 9 (41) | $10,575 | $12,235 | $8,176 | $6,726 | | $9,781 | $6,591 |
| Musculoskeletal | 4 (50) | 4 (50) | $11,981 | $7,894 | $16,068 | $12,296 | | $7,145 | $14,493 |
| Nervous | 3 (50) | 3 (50) | $12,309 | $9,665 | $14,947 | $11,246 | | $7,151 | $18,802 |
| Pregnancy | 5 (50) | 5 (50) | $12,870 | $17,678 | $8,062 | $9,609 | | $9,737 | $7,924 |
| Injury & Poisoning | 14 (50) | 14 (50) | $16,004 | $16,546 | $15,462 | $14,717 | | $14,170 | $16,029 |
| Circulatory | 27 (51) | 26 (49) | $17,841 | $21,641 | $13,738 | $14,674 | | $17,178 | $10,171 |
| Infectious | 4 (50) | 4 (50) | $19,137 | $16,612 | $21,663 | $8,821 | | $5,632 | $18,689 |
| Neoplasms | 3 (50) | 3 (50) | $34,871 | $37,605 | $32,136 | $30,425 | | $21,242 | $34,089 |

**Additional File 3.** Mean and median costs of index hospitalization by indication for subjects who

continued to smoke versus those who were abstinent at 6-month follow-up, according to diagnostic category

**Supplemental Table 3B.** Mean and median 6-month follow-up costs by indication for subjects who

continued to smoke versus those who were abstinent at 6-month follow-up

| Indication | Abstainer*  N (%) | Non-Abstainer  N (%) | Mean | | | Median | | |
| --- | --- | --- | --- | --- | --- | --- | --- | --- |
|  |  |  | **All** | **Abstainer*** | **Non-Abstainer** | **All** | **Abstainer*** | **Non-Abstainer** |
| Other | 1 (25) | 3 (75) | $35,654 | $46,486 | $32,043 | $29,041 | $46,486 | $11,596 |
| Genitourinary | 1 (50) | 1 (50) | $10,265 | $6,361 | $14,107 | $10,265 | $6,361 | $14,107 |
| Skin | 4 (57) | 3 (43) | $15,851 | $9,882 | $23,809 | $6,968 | $6,444 | $13,060 |
| Respiratory | 11 (52) | 10 (48) | $8,405 | $5,100 | $12,041 | $2,705 | $1,814 | $5,186 |
| Endocrine | 3 (50) | 3 (50) | $44,787 | $25,466 | $64,107 | $10,913 | $21,334 | $493 |
| Mental Illness | 6 (35) | 11 (65) | $18,463 | $24,386 | $15,232 | $6,378 | $13,674 | $6,378 |
| Digestive | 13 (59) | 9 (41) | $8,621 | $10,105 | $6,477 | $4,410 | $6,547 | $3,816 |
| Musculoskeletal | 4 (50) | 4 (50) | $2,131 | $2,131 | $5,133 | $1,057 | $1,057 | $2,062 |
| Nervous | 3 (50) | 3 (50) | $19,091 | $20,604 | $17,578 | $16,931 | $17,364 | $2,043 |
| Pregnancy | 5 (50) | 5 (50) | $7,440 | $9,474 | $5,405 | $5,546 | $8,172 | $2,920 |
| Injury & Poisoning | 14 (50) | 14 (50) | 15,919 | $18,509 | $13,330 | $9,431 | $11,734 | $6,987 |
| Circulatory | 27 (51) | 26 (49) | $18,886 | $45,532 | $19,258 | $12,260 | $11,732 | $13,901 |
| Infectious | 4 (50) | 4 (50) | $9,326 | $13,092 | $5,561 | $4,987 | $12,444 | $4,565 |
| Neoplasms | 3 (50) | 3 (50) | $61,617 | $36,518 | $86,716 | $48,801 | $39,659 | $57,944 |

*Self-reported abstinence at 6 months after hospital discharge.
